# The Emerging Role of Pathogenesis of IgA Nephropathy

**DOI:** 10.3390/jcm7080225

**Published:** 2018-08-20

**Authors:** Meng-Yu Wu, Chien-Sheng Chen, Giou-Teng Yiang, Pei-Wen Cheng, Yu-Long Chen, Hsiao-Chen Chiu, Kuan-Hung Liu, Wen-Chin Lee, Chia-Jung Li

**Affiliations:** 1 Department of Emergency Medicine, Taipei Tzu Chi Hospital, Buddhist Tzu Chi Medical Foundation, New Taipei City 231, Taiwan; skyshangrila@gmail.com (M.-Y.W.); holeyeye@yahoo.com.tw (C.-S.C.); gtyiang@gmail.com (G.-T.Y.); yulong0129@gmail.com (Y.-L.C.); 2 Department of Emergency Medicine, School of Medicine, Tzu Chi University, Hualien 970, Taiwan; 3 Yuh-Ing Junior College of Health Care & Management, Kaohsiung 807, Taiwan; pwcheng@vghks.gov.tw; 4 Department of Medical Education and Research, Kaohsiung Veterans General Hospital, Kaohsiung 813, Taiwan; 5 Department of Obstetrics and Gynecology, Taipei Tzu Chi Hospital, Buddhist Tzu Chi Medical Foundation, New Taipei City 231, Taiwan; 97311141@tzuchi.com.tw; 6 Department of Obstetrics and Gynecology, School of Medicine, Tzu Chi University, Hualien 970, Taiwan; 7 Division of Nephrology, Department of Internal Medicine, National Cheng Kung University Hospital, College of Medicine National Cheng Kung University, Tainan 704, Taiwan; drkuanhungliu@gmail.com; 8 Division of Nephrology, Department of Internal Medicine, Chang Bing Show Chwan Memorial Hospital, Changhua 505, Taiwan; 9 Research Assistant Center, Show Chwan Memorial Hospital, Changhua 500, Taiwan

**Keywords:** inflammation, IgA nephropathy, anti-glycans autoantibody, galactose-deficient IgA1, mesangio-podocytic-tubular crosstalk

## Abstract

IgA nephropathy is an autoimmune disease induced by fthe ormation of galactose-deficient IgA1 and anti-glycans autoantibody. A multi-hit hypothesis was promoted to explain full expression of IgA nephropathy. The deposition of immune complex resulted in activation of the complement, increasing oxidative stress, promoting inflammatory cascade, and inducing cell apoptosis via mesangio-podocytic-tubular crosstalk. The interlinked signaling pathways of immune-complex-mediated inflammation can offer a novel target for therapeutic approaches. Treatments of IgA nephropathy are also summarized in our review article. In this article, we provide an overview of the recent basic and clinical studies in cell molecular regulation of IgAN for further treatment interventions.

## 1. Introduction

IgA nephropathy (IgAN), also known as Berger’s disease, is the most common primary glomerulonephritis that occurs when immunoglobulin A (IgA) deposits in the kidneys lead to the development of local inflammation and renal injury. IgA deposits are also associated with other systemic diseases, such as Henoch-Schönlein purpura (HSP), which is considered the systemic IgA vasculitis affecting skin, joints, gut, and kidneys. In up to 50% of IgAN patients, this disease progresses to the end-stage renal disease within 20 to 25 years [1], but, according to the currently available data, kidneys are considered innocent bystanders [2]. Further transplantation data has demonstrated that IgA deposits in kidneys from donors with IgAN are often decreased, but they are still present in allografts. These IgA molecules predominantly belong to the polymeric IgA1 subclass, and they are aberrantly glycosylated. Other proteins, such as IgG and complement 3 (C3), have been reported to be deposited as well, and may contribute to disease severity [3]. Although this disease has been studied for years, the detailed molecular regulation of IgAN pathophysiology remains unclear, especially concerning antibody production and immune response. In this review, we summarized the growing literature on the disease pathogenesis, spanning form basic to clinical studies, to provide an overview of IgAN microenvironment and renal inflammation and to help identify novel treatment approaches.

## 2. Epidemiologic and Clinical Characteristics of IgAN

IgAN is a common cause of chronic kidney disease, with an overall incidence of at least 2.5 per 100,000 individuals [4]. Epidemiological studies have demonstrated that the incidence is increased in individuals of Pacific Asian origin, which also show an increased risk of developing the end-stage renal disease [5,6]. IgAN more frequently occurs in males than in females, at a ratio of 2:1 to 6:1 [7], and usually affects 16–35 year-olds, which account for approximately 80% of total patients. According to the clinical presentation, the disease can be divided into episodic gross hematuria and persistent microscopic hematuria. Macroscopic hematuria occurs in 40% to 50% of patients within only 1 or 2 days after an upper respiratory tract infection, unlike post-streptococcal glomerulonephritis, which occurs within weeks. Other infections, such as urinary tract infection and acute gastrointestinal infection, are less commonly the initiating events. Asymptomatic microscopic hematuria occurs in 20–30% patients, and it is accompanied by mild proteinuria (less than 2 g/day). Nephrotic syndrome and acute renal failure are less common, occurring in only 5% of IgAN patients. Nephrotic syndrome is characterized by proteinuria, edema, dyslipidemia, and hypoalbuminemia, while some studies reported the coexistence of IgAN and minimal change disease [2,8]. In a cohort study, only 2.57% IgAN patients were diagnosed with diffuse IgAN with minimal changes [9]. Acute renal failure may be induced by severe inflammatory response leading to the crescent formation and development of crescentic IgAN. Additionally, it can be induced by severe hematuria as well, leading to tubular occlusion. Renal biopsy is the gold standard for the establishment of IgAN diagnosis, and, using immunofluorescence staining, IgA deposits with mesangial proliferation are characteristically observed.

The pathologic findings can provide not only the histological diagnosis of IgAN but also help predict the clinical outcomes as well. In 2009, the Oxford classification of IgAN, based on the characteristic findings underlying IgAN pathogenesis, was published [10]. Four key pathologic findings were reported: mesangial hypercellularity, endocapillary hypercellularity, segmental glomerulosclerosis, and tubular atrophy and interstitial fibrosis (Table 1). Together, these characteristics allow clinical predictions according to the MEST or Oxford score.

The applicability of Oxford score was validated in several large studies [11,12]. The VALIGA cohort study showed that the histologic features of IgAN patents are independently correlated with the clinical outcomes after adjusting for clinical baseline data, including mesangial proliferation, shown to be associated with the increased risk of proteinuria, and high tubular atrophy and interstitial fibrosis score, shown to be associated with poor renal outcome. The Oxford score has been shown to provide a timely and accurate prediction of the IgAN outcome. Recently, the role of crescent formation was demonstrated to be important as well. Early data suggest that the presence of crescents in more than 50% of glomeruli increases the risk of end-stage renal disease [13]. The crescent formation scores were included in the prediction of the clinical outcome of IgAN (Table 1) in a meta-analysis study [14], showing that the presence of crescents increases the risk of poor renal prognosis. Although, by using the Oxford score, we can predict disease progression in a minor population of IgAN patients, several other factors have been strongly associated with the disease outcome.

## 3. Pathogenesis of IgAN

**Figure 1:**
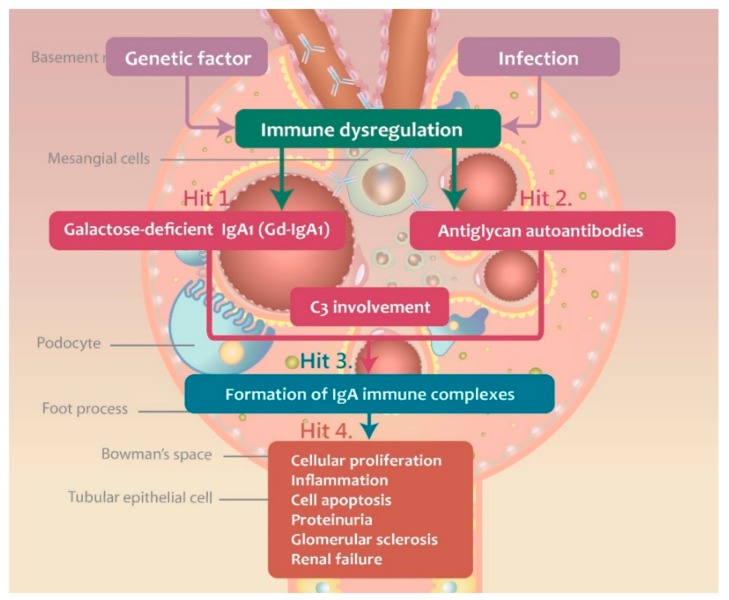
The multi-“hit” hypothesis explaining the immunopathogenesis of IgAN.

Currently, IgAN is considered an autoimmune disease, with a multi-hit hypothesis proposed to explain its immunopathogenesis [15]. The production of galactose-deficient IgA1 (Gd-IgA1) is the first hit during the development of IgAN, and it plays an important role in the formation of immune complexes. The second hit represents the production of IgG autoantibodies, which target the O-glycans in the hinge region, leading to the formation of immune complexes. These complexes induce local inflammatory responses and they are deposited in kidneys, leading to the activation and mesangial cell damage. In Figure 1, the steps of the multi-hit IgAN development are presented. The three major antibodies, Gd-IgA1, antiglycan autoantibody (anti-Gd-IgA1 autoantibody), and C3 are involved in the IgAN pathogenesis.

### 3.1. Gd-IgA1 Signaling and Production

**Figure 2:**
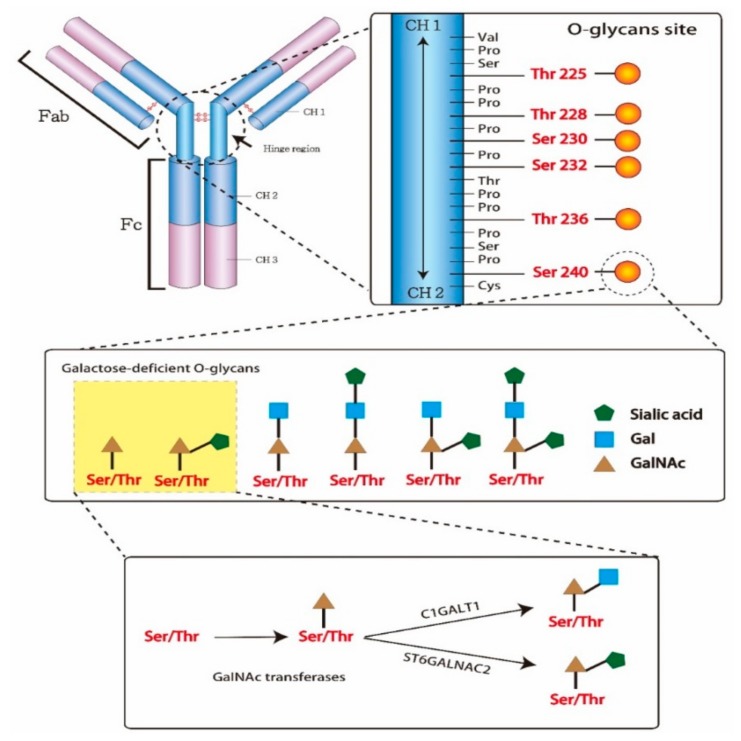
Structure of immunoglobulin A1 (IgA1) hinge region with O‑glycans. In the hinge region, IgA1 potentially has three to six O-glycans. The O-glycan attached to IgA1 via the link with serine/threonine residues and oxygen atoms. The structure of O-glycans consisted of *N*-acetylgalactosamine (GalNAc), galactose and/or sialic acid. The attachment of GalNAc to serine/threonine residues by GalNAc transferases is first step of glycosylation. Second step, the GalNAc can be added galactose by glycoprotein *N*-acetylgalactosamine 3-β-galactosyltransferase (C1GALT1) or sialic acid by α-*N*-acetylgalactosaminide α2,6-sialyltransferase 2 (ST6GALNAC2).

An antibody is a Y-shaped glycoprotein produced by B-cells and plasma cells to neutralize external pathogens. It consists of two identical heavy and light chains each. The antigen-binding sites in the arm of antibodies is designated as the fragment of the antigen-binding region (Fab), while the base arm of antibodies, contributing to the regulation of immune cell activity, is the crystallizable region (Fc). The Fc can activate several types of cells and inflammatory response by binding complement proteins and other immune molecules. In IgAN patients, Gd-IgA1 is produced due to an abnormal biosynthesis of O-glycans, which are attached to the heavy chain through serine/threonine residues and oxygen atoms. The nine sites for glycan attachment in the hinge region of the IgA1 heavy chain are usually one-third or two-thirds glycosylated. O-glycans consist of *N*-acetylgalactosamine (GalNAc), galactose, and/or sialic acid. The attachment of GalNAc to serine/threonine residues through the activity of GalNAc transferases is the first step in the glycosylation process. Afterward, galactose and sialic acid can be added to the GalNAc by glycoprotein *N*-acetylgalactosamine 3-β-galactosyltransferase (C1GALT1) and α-*N*-acetylgalactosaminide α2,6-sialyltransferase 2 (ST6GALNAC2), respectively (Figure 2). However, the GalNAc modified by sialic acid cannot be galactosylated. Gd-IgA1 molecules show a higher avidity toward mesangial cells, which leads to local inflammation. The overexpression of enzymes involved in glycosylation, such as glycosyltransferases, plays a critical role in G-d IgA1 overproduction [16,17], but the overexpression of ST6GALNAC2 and downregulation of C1GALT1 expression may promote G-d IgA1 production as well. Additionally, environmental factors may affect its production as well, together with different cytokines and microRNAs that may increase enzyme activity imbalance and aberrant gene expression [16,17,18,19,20,21]. Furthermore, the oversialylation of G-d IgA1 provides it with a strong negative charge, which is difficult to metabolize by the liver and increases the affinity of binding to the mesangial cells [22].

Production of Gd-IgA1 may be induced by the combination of genetic and environmental factors. In a study investigating 64 family members of IgAN patients, Gd-IgA1 serum levels were shown to be increased in 25% of these family members [23]. However, synpharyngitic hematuria in patients with IgAN represents a renal complication driven by the environmental factors, which induce the production of Gd-IgA1 in mucosal tissues. B-cells were shown to play a crucial role in IgAN development, by exhibiting increased IL-4 and IFN-γ production upon the exposure to pathogen [24], which also leads to the activation of the antigen-presenting cells, such as monocytes and neutrophils, and they express Toll-like receptors (TLRs), binding pathogen-associated molecular patterns (PAMPs) and danger-associated molecular patterns (DAMPs), such as bacterial proteins, RNAs, and DNAs [25,26]. Additionally, they can recruit other immune cells by releasing chemoattractants and chemokines.

Furthermore, endothelial and epithelial cells express increased levels of adhesion molecules, such as intercellular adhesion molecule-1 (ICAM-1) and vascular-cell-adhesion molecule-1 (VCAM-1) [27]. Following the uptake of pathogens and other antigens, monocyte mature and differentiate into macrophages, which then produce IL-1, IL-6, IL-12, IL-15, IL-18, TNF-α, migration inhibitory factor, and transforming growth factor (TGF)-β. The dendritic cells can also release a variety of pro-inflammatory mediators, including IL-6, TNF, IL-12, IL-23, and granulocyte macrophage colony-stimulating factor (GM-CSF). These pro-inflammatory factors recruit T-cells and B-cells, which are activated after the interactions with co-stimulatory molecules, such as CD40, OX40L, CD80/86, and CD28, and induce the activation of the inflammatory cascade. Increased serum cytokine levels promote IgA antibody release from B-cells.

**Figure 3:**
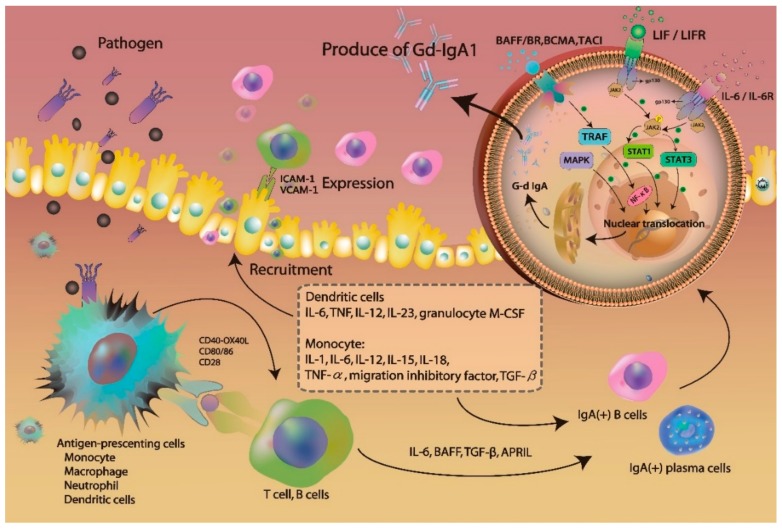
The mechanism of activating B cells to produce the galactose-deficient IgA1.

IL-6 was reported to be associated with G-d IgA1 production [28,29,30]. In vitro, IL-6 binding to the IL-6 receptor promotes Janus kinase 2 (JAK2) phosphorylation to activate signal transducer and activator transcription 3 (STAT3), leading to its nuclear translocation (Figure 3) [30]. Additionally, a prolonged exposure to IL-6 in IgAN model was shown to be associated with the increase in STAT3 activation and IgA1 production [29,31]. STAT3 activation leads to the increase in Gd-IgA1 production as well, through the induction of specific glycosyltransferase expression. Leukemia inhibitory factor (LIF) is an IL-6-class cytokine, which binds to gp130 and LIF receptor (LIFR) to activate JAK2, leading to STAT1/3 activation and nuclear translocation. B-cell-activating factor (BAFF), a cytokine belonging to the tumor necrosis factor (TNF) ligand family, can activate multiple receptors, including BAFF receptor (BR), B-cell maturation antigen (BCMA), and transmembrane activator and calcium-modulating/cyclophilin ligand protein (TACI) [32,33,34]. TNF-receptor-associated factor (TRAF) activates NF-kB and induces its nuclear translocation. Additionally, mitogen-activated protein kinase (MAPK) activation was shown to affect O-glycosylation as well. Finally, nuclear translocation of STATs and NF-kB promotes Gd-IgA1 production in B-cells and plasma cells (Figure 3).

T-cells play an important role in G-d IgA1 production, as they release APRIL, a proliferation-inducing ligand encoded by *TNFSF13* located on chromosome 17p13 [35,36], which promotes lymphocyte proliferation and IgA class switching. IgAN patient studies demonstrated that the expression of APRIL is increased in these patients, compared with that in the control group [37,38]. In vitro studies showed that BAFF and APRIL have overlapping functions and receptors, and they both lead to the formation of IgA deposits [36,39]. Therefore, APRIL, BAFF, LIF, and IL-6 may represent novel therapeutic targets for the reduction of serum IgA levels and IgA deposition.

After exposure to pathogens, the host initiates’ innate and adaptive immune responses were activated. The expression of toll-like receptors (TLRs) on antigen-presenting cells, such as monocyte/macrophage dendritic cells, and neutrophil, recognized pathogens and released several proinflammatory cytokines. The production of cytokines and chemokines recruited other immune cells to the infection site. The expression of IL-6, BAFF, TGF-β and APRIL triggered the activation of IgA-secreting cells, such as B cells and plasma cells. The IL-6 binding the IL-6 receptor promotes Janus kinase 2 (JAK2) phosphorylation to activate signal transducer and activator transcription 3 (STAT3). Leukemia inhibitory factor (LIF) binds gp130 and LIF receptor (LIFR) to activate JAK2, leading to STAT1/3 activation and nuclear translocation. B-cell-activating factor (BAFF) activated multiple receptors, such as BAFF receptor (BR), B-cell maturation antigen (BCMA), and transmembrane activator and calcium-modulating/cyclophilin ligand protein (TACI) causing NF-kB activation and nuclear translocation. Mitogen-activated protein kinase (MAPK) was also activated. The nuclear translocation of the STATs and NF-kB promoted production of Gd-IgA1 in B cells and plasma cells.

### 3.2. Immune Complex Deposition-Associated Induction of Inflammation and Complement Activation

Anti-Gd-IgA1 antibodies further induce IgAN development. Previously, immune complexes were shown to consist of the polymeric Gd-IgA1 and IgG antibodies [40,41]. In HSP without nephritis patients, serum antibody analysis identified only IgA antibodies against IgA immune complexes, unlike in patients with HSP and with nephritis, which were shown to have IgA antibodies against IgA and IgG immune complexes [40,42,43,44]. The presence of IgG antibodies targeting GalNAc residues in the hinge region O-glycans of IgA1 heavy chain was confirmed using ELISA [41,45]. Complementarity-determining region 3 (CDR3) at the heavy chains of anti-Gd-IgA1 antibodies was shown to play an important role in immune complex formation [41,46,47], and its structure; specifically, the third amino acid, was shown to differ between IgAN patients and healthy controls (serine and alanine, respectively), providing an increase in its affinity toward Gd-IgA1 [15,47,48]. Anti-Gd-IgA1 antibody levels were shown to be associated with IgAN progression, severity, and prognosis [41,49].

**Figure 4:**
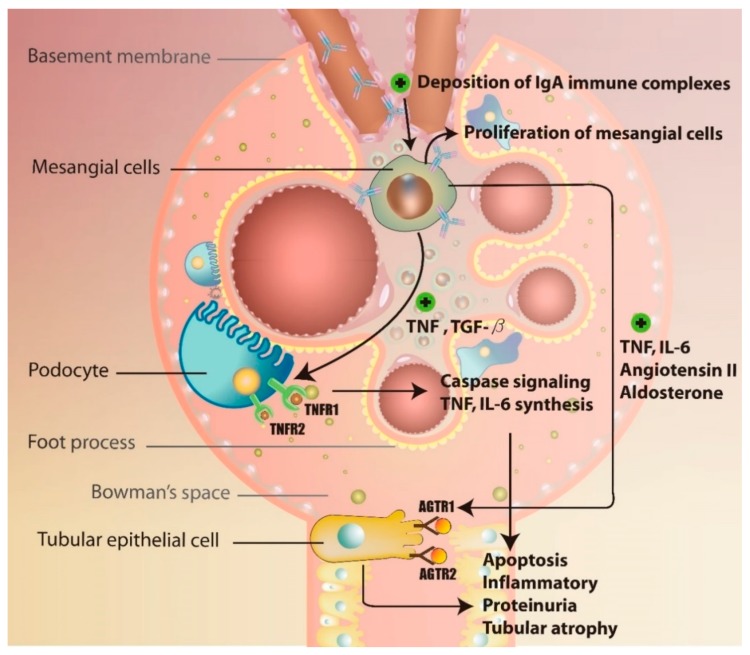
The mechanism of glomerulo-podocytic-tubular crosstalk in IgAN.

The detailed mechanisms underlying the formation of anti-Gd-IgA1 autoantibody remain unclear. Currently, it is believed that the production of this antibody may be induced by prior viral and/or bacterial infections, such as those induced by Epstein-Barr virus and Streptococcus spp. Based on the investigations of post-upper-respiratory-tract-infection macroscopic hematuria, molecular mimicry theory was introduced, explaining the production of anti-Gd-IgA1 antibodies due to the aberrant production of microbial-specific IgG antibodies, leading to the formation of immune complexes and their deposition in mesangial cell [50,51]. The binding of IgA immune complexes further activates mesangial cells to produce pro-inflammatory factors, such as TNF, TGF-β, IL-6, and angiotensin II (Ang II), leading to alterations in the glomerular permeability and subsequent proteinuria and tubulointerstitial injury [52,53]. A previous study showed that the IgA1 molecules isolated from the asymptomatic relatives of IgAN patients may induce mesangial cell activation as well [54]. IgAN increases the expression of receptor-type tyrosine-protein phosphatase C in lymphocytes and IL-2 receptor subunit-α in monocytes, promoting inflammatory changes in renal tubular cells. The activation of inflammatory cascade induces tubulointerstitial injury and renal fibrosis via glomerulo-podocytic-tubular crosstalk, while TNF release promotes the expression of TNF receptor 1 and 2 (TNFRSF1A and TNFRSF1B) in podocytes [55]. TNF-TNFR1 interactions further induce IL-6 expression and cell apoptosis, while TNF-TNFR2 interactions were shown to induce inflammatory responses [52,53]. Following the podocyte apoptosis, the proteinuria and tubular atrophy develop in the IgAN patients, together with the accumulation of TNF from mesangial cells and podocytes, which also induced tubular epithelial cell damage. Ang II released from mesangial cells was shown to interact with the angiotensin receptor type 1 and 2 (AGTR1 and 2) in tubular epithelial cells [56,57,58]. Ang II-AGTR1 binding was demonstrated to activate inflammatory cascade by upregulating protein kinase C and MAPK pathways, and Ang II-AGTR2 interactions were shown to lead to the activation of cell apoptosis through caspase signaling. Aldosterone released by mesangial cells induces renal tubular epithelial cells apoptosis as well [58]. Together, this leads to tubular atrophy and may progress to renal failure [59] (Figure 4). Finally, the release of TGF-β induces glomerular fibrosis through the expression of connective tissue growth factor (CTGF) via sphingosine 1-phosphate receptor 5 (S1P5) [60,61].

### 3.3. Complement Activation during IgAN Pathogenesis

**Figure 5:**
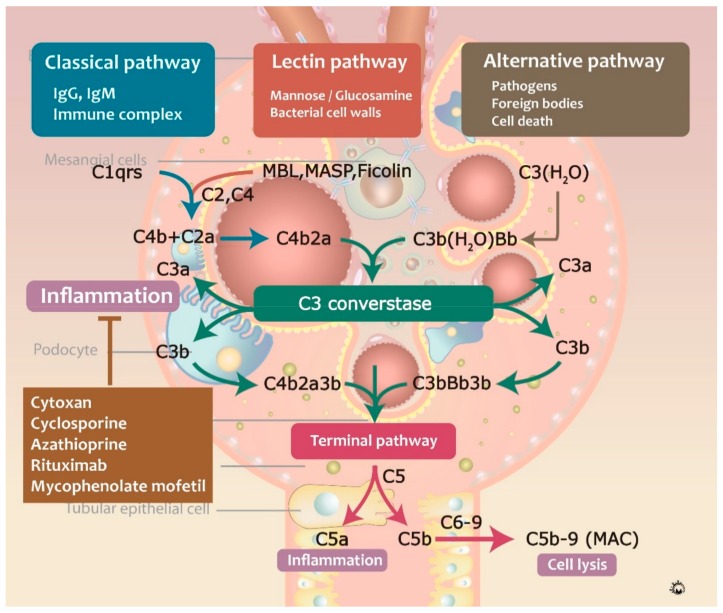
The mechanism of complement activation pathways in IgAN.

Proteomic and immunologic analyses revealed that C3 is frequently involved in the formation of circulating immune complexes and immunodeposits in IgAN patients [62,63]. C3 in immune complexes consists of α and β chains, together with other C3 components, such as iC3b, C3c, and C3dg. Complementary activation can occur through three major pathways: classical, lectin, and alternative pathways. The classical complement pathway was shown to be activated by the formation of antigen-antibody complexes, consisting of C1q protein against IgG, IgM, bacterial and viral surface proteins, apoptotic cells, and acute phase proteins [64]. Activated C1q binds to C1r and C1s, cleaving C2 and C4 into C4b2a complex, a C3-convertase, which further cleaves C3 into C3a and C3b, and induced the formation of C5-convertase (C4bC2aC3b) [65]. Afterward, C5 convertase cleaves C5 into C5a and C5b, and C3a and C5a are involved in the recruitment of leukocytes. Additionally, C5b binds to C6, C7, C8, and C9, forming the membrane attack complex (MAC) and leading to target cell membrane lysis. In contrast, alternative pathway activation can be triggered by C3b protein-binding pathogens, foreign bodies, and cell death. C3 is initially spontaneously hydrolyzed to form C3(H2O) and binds to factor Bb, forming C3(H_2_O)Bb, a C3-convertase [66]. This reaction is mediated by several factors, including complement factors I, H and D, properdin, and DAF. The lectin pathway is activated by mannose or glucosamine on the bacterial cell walls via mannose-binding lectin (MBL) [67], in the process similar to the activation of the classical complement pathway and C4b2a generation (Figure 5) [68].

IgA-based activation of alternative complement pathway plays a critical role in the IgAN pathogenesis, and C3 activation can be detected by screening for the degraded components of C3, since they are crucial for the activation of a complement through mediating factors I and H. Recently, genome-wide association studies identified a single nucleotide polymorphism (SNP) at 1q32 in factor H gene, which was shown to have protective effects against IgAN pathogenesis [37,69]. The deletion of complement factor H-related (CFHR) genes one and three was in linkage disequilibrium with the identified SNP [62] and it was associated with a decrease in C3 mesangial deposition in IgAN [70]. The deposition of terminal complement complex was shown to coincide with its increased urinary excretion [71,72,73] and induced mesangial stress and podocyte damage, which leads to the release of fibronectin, TGF-β, and IL-6 [74,75,76,77]. Furthermore, serum C3 levels can be used as a disease biomarker, while the IgA/C3 ratio is an indicator of disease severity [78,79].

## 4. Therapeutic Strategies for the Treatment of IgAN

In treatment of IgAN, the patient selection for therapy is important and based on the risk of progressive kidney disease. The assessment of blood pressure, serum creatinine, proteinuria, and estimated glomerular filtration rate in IgAN patients is necessary. The IgAN patients with isolated hematuria, or with minimal proteinuria (less than 500 mg/day), and normal eGFR are at low risk of renal failure. These populations may not disgnose IgAN due to not receiving a renal biopsy. The follow up for 1 year is recommended. The IgAN patients with proteinuria (above 500 mg/day), or slightly slowly reducing GFR, and mild to moderate severity of histologic findings are recommended for non-immunosuppressive therapies. The severe or rapidly progressive proteinuria or renal biopsy with severe histologic findings may be suggested to treat. In IgAN patients with low risk, the diet, fish oil, and ACEi or sartans are alternatives for anti-inflammation to control disease progression. In IgAN patients with moderate or high risk, it is important to prevent the formation of Gd-IgA1and anti-Gd-IgA1 autoantibodies. The management of IgAN includes anti-inflammatory and immunosuppressive therapies, such as corticosteroids, mycophenolate mofetil (MMF), rituximab, cyclophosphamide, and azathioprine, and blood pressure lowering agents, including angiotensin receptor blockers and calcium channel blockers [80,81]. A number of pharmacologic immunosuppressive therapies have been evaluated for the use in IgAN treatment, many of them were shown to have partial effects in different patient subgroups.

### 4.1. Non-Immunosuppressive Therapies

In IgAN patients with low risk of disease progression, the non-immunosupressive therapies were suggested as first line therapy for preventing disease progression, such as diet control, fish oil, Angiotensin-converting enzyme (ACE) inhibitors, angiotensin II receptor blockers (ARB) or sartans. The angiotensin-converting enzyme plays an important role in regulating vasoconstriction and vessel permeability [82]. ACEI or ARB regulated systemic blood pressure, preventing hypertension and hyper-intraglomerular pressure, and inhibiting the progression of most proteinuric chronic kidney diseases. Therefore, ACEI controlled both proteinuria and secondary glomerular damage. A meta-analysis reported by J. Cheng et al. [83] included seven RCTs (585 patients) and revealed ACEI/ARB therapies significantly protecting renal function and reducing proteinuria. In addition, the angiotensin II is a powerful proinflammatory mediator by stimulating angiotensin II receptor type 1 (AT1R), inducing integrins, adhesion molecules, cytokines and growth factor via redox-sensitive pathways, leading to vascular inflammation [84,85]. The ACEI/ARB had an anti-inflammatory effect of preventing vascular remodeling and local inflammation, leading to decreased proteinuria and disease progession.

The Eicosapentaenoic acid (EPA) and docosahexaenoic acid (DHA) are n-3 fatty acids in fish oil and present the anti-inflammatory effect via multiple potential mechanisms [86]. EPA and DHA generated resolvins and protectins via cyclooxygenase and lipoxygenase enzymes, which regulated inflammation responses [87,88]. In previous reports, the resolvin and protectin in vitro inhibit neutrophilic infiltration by decreasing the activity of migration, and thereby preventing local inflammation [89]. The resolvin and protectin also inhibit the production of IL-1β, TNF and IL-1β, which are factors that may promote inflammatory caspase and the release of other cytokines [88]. In vivo, the role of fish oil and omega-3 fatty acids in the treatment of IgA nephropathy is still investigated [90]. A meta-analysis article was reported by Sharon Reid et al. [91] including 56 randomised controlled trials of non-immunosuppressive agents including a total of 2838 IgAN patients. The results showed no overall beneficial effect on renal outcomes, such as serum creatinine, creatinine clearance, and proteinuria. Although the benefit from fish oil has not been significantly established, no significant side effect was reported. It seems that fish oil can be tried in IgAN patients with low risk of renal failure for preventing disease progression.

### 4.2. Corticosteroids

For the control of IgAN progression, anti-inflammatory drugs are crucial. In vitro studies have demonstrated that corticosteroids decrease the production of prostaglandin by activating annexin I and inhibiting the production of arachidonic acid [92,93]. The activation of MAPK phosphatase 1 was shown to inhibit cytosolic phospholipase A2a by decreasing the activity of MAPKs and MAPK-interacting kinase. Additionally, corticosteroids inhibit c-Jun, Fos, and NF-κB pathways, consequently inhibiting inflammatory responses. Due to these corticosteroid effects, they are widely used as in the treatment of autoimmune diseases [94]. Early studies reported that corticosteroids may improve renal outcomes in these diseases, showing that patients treated with corticosteroids have improved 5-year renal survival and reduced proteinuria, compared with patients treated with supportive therapy [95]. The results of a randomized trial demonstrated that corticosteroids combined with angiotensin-converting enzyme inhibitor improved renal outcome, preventing end-stage renal disease and the doubling of serum creatinine over 8 years [96,97]. Combination therapy, including corticosteroids and an immunosuppressive agent, has been used for the treatment of advanced progressive IgAN or patients with increased risk of IgAN, including those with hypertension and elevated serum creatinine [98].

However, the adverse effects of combination therapy in IgAN patients have been reported in several studies [99,100]. The results of large studies demonstrated that the application of corticosteroids in patients with proteinuria at above 3 g/day must be closely monitored to prevent the development of side effects. In IgAN patients with proteinuria and excretion levels higher than 1 g/day, no significant benefits of corticosteroid use were observed, while for the IgAN patients with the excretion rate from 1–3 g/day, currently available data are insufficient to determine the potential benefits of corticosteroid application. The modified oral forms of corticosteroids, such as budesonide, which target the mucosal lymphoid tissue in the ileocecal region and regulates IgA production, was shown to reduce the side effects associated with the application of intravenous corticosteroids. Theoretically, the concentration of budesonide considerably decreases before entering blood, which decreases systemic side effects [101]. A recent phase 2 placebo-controlled trial confirmed a significant reduction of proteinuria in patients treated with budesonide, but its efficacy and safety should be further confirmed.

### 4.3. Mycophenolate Mofetil

MMF, an inhibitor of inosine-5′-monophosphate dehydrogenase involved in de novo synthesis of guanosine nucleotides, acts by depleting these nucleotides to inhibit T- and B-lymphocyte proliferation, inflammatory cascade activation, and the formation of antibodies [102,103]. MMF additionally inhibits glycosylation, decreases the expression of adhesion molecules for the recruitment of immune cells, and decreases nitric oxide production and oxidative stress [104]. Due to these functions, MMF has strong anti-inflammatory activity in IgAN therapy [105]. Furthermore, MMF shows no renal toxicity, it does not promote fibrotic side effect, and it does not increase blood pressure, and therefore, it has been widely used for the treatment of autoimmune disorders [80,106]. A randomized controlled trial, including 40 patients with proteinuria at above 1 g/day, showed that the severity of proteinuria decreased in IgAN patients treated with MMF, while increasing renal survival [107,108]. However, the conclusions of these studies are limited by the small sample size [109], and another randomized placebo-controlled clinical trial, performed in patients with proteinuria, was stopped early, due to the lack of observation of any considerable benefits [110]. In patients with advanced IgAN, renal outcome was shown to be aggravated following the treatment with MMF. Therefore, currently, the regular use of MMF is not recommended for the IgAN treatment [111], but further studies are necessary to obtain a definite conclusion.

### 4.4. B-cell Depletion/Inhibition

Blisibimod is a selective antagonist of BAFF that promotes the proliferation and differentiation of B-cells, leading to the development of B-cell autoimmune diseases, such as lupus nephritis and IgAN. Blisibimod suppressed the activity of BAFF, inhibiting the activation of B-cells and improving disease outcomes [112]. However, the safety and efficacy of blisibimod are currently being examined in clinical trials. Spleen tyrosine kinase mediates the maturation and proliferation of the B-cell lineage. In IgAN, the overexpression of phosphorylated SYK was observed in renal biopsies [105], while the pharmacological inhibition of its expression led to a significant inhibition of B-cell proliferation [105], together with the human mesangial cell involvement in inflammatory processes. Since 2014, the efficacy and safety of fostamatinib, a selective oral SYK inhibitor, has been investigated in an international multicenter study [113].

Taken together, although several therapeutic approaches have been reported, all of them showed controversial effects or only partial improvements in IgAN patients. Therefore, future randomized controlled trials are necessary to determine the best IgAN treatment strategies.

## 5. Conclusions

IgAN is a glomerular autoimmune disease, developing due to the formation of Gd-IgA1 and anti-glycan autoantibodies. A multi-hit hypothesis, aimed at explaining its origins and mechanisms underlying the pathogenesis of this disease, suggests that the deposition of immune complexes results in the complement activation, increasing oxidative stress, promoting inflammatory cascade activation, and inducing cell apoptosis. Here, we provided an overview of the recent basic and clinical studies examining the regulation of molecular pathways underlying IgAN pathogenesis, including the formation of Gd-IgA1 and anti-glycan autoantibodies, activation of complement, and the induction of inflammation via mesangio-podocytic-tubular crosstalk. Finally, although the detailed mechanisms of IgAN development are not fully elucidated yet, we believe that the complete understanding of the pathophysiology of IgAN may provide a strong foundation for the development of novel therapeutic targets.

## Figures and Tables

**Table 1:** Modified Oxford classification and MEST score system (adapted from Roberts, I. S. et al. [10]).

Histological Finding	Score
Mesangial hypercellularity	M0: Presence of mesangial hypercellularity in <50% glomeruli M1: Presence of mesangial hypercellularity in >50% glomeruli
Endocapillary hypercellularity	E0: No endocapillary hypercellularity E1: Presence of any endocapillary hypercellularity
Segmental glomerulosclerosis	S0: No segmental glomerulosclerosis S1: Presence of any segmental glomerulosclerosis
Tubular atrophy and interstitial fibrosis	T0: 0–25% tubular atrophy/interstitial fibrosis in cortical area T1: 26–50% tubular atrophy/interstitial fibrosis in cortical area T2: >50% tubular atrophy/interstitial fibrosis in cortical area
Cellular or fibrocellular crescents	C0: no cellular or fibrocellular crescents C1: Presence of cellular/fibrocellular crescents in <25% glomeruli C2: Presence of cellular/fibrocellular crescents in >25% glomeruli

M: mesangial hypercellularity; E: endocapillary hypercellularity; S: segmental glomerulosclerosis; T: tubular atrophy and interstitial fibrosis; C: crescent formation.

## References

[1] Geddes C.C. (2003). A Tricontinental View of IgA Nephropathy. Nephrol. Dial. Transplant..

[2] Silva F.G. (1982). Disappearance of glomerular mesangial IgA deposits after renal allograft transplantation. Transplantation.

[3] Suzuki H. (2011). The Pathophysiology of IgA Nephropathy. J. Am. Soc. Nephrol..

[4] McGrogan A., Franssen C.F., De Vries C.S. (2011). The incidence of primary glomerulonephritis worldwide: A systematic review of the literature. Nephrol. Dial. Transplant..

[5] Barbour S.J. (2013). Individuals of Pacific Asian origin with IgA nephropathy have an increased risk of progression to end-stage renal disease. Kidney Int..

[6] Barbour S.J. (2016). The MEST score provides earlier risk prediction in lgA nephropathy. Kidney Int..

[7] Wyatt R.J. (1998). Epidemiology of IgA nephropathy in central and eastern Kentucky for the period 1975 through 1994 Central Kentucky Region of the Southeastern United States IgA Nephropathy DATABANK Project. J. Am. Soc. Nephrol..

[8] Lai K.N. (1986). An overlapping syndrome of IgA nephropathy and lipoid nephrosis. Am. J. Clin. Pathol..

[9] Li X.W. (2016). Long-term outcome of IgA nephropathy with minimal change disease: A comparison between patients with and without minimal change disease. J. Nephrol..

[10] Roberts I.S. (2009). The Oxford classification of IgA nephropathy: Pathology definitions, correlations, and reproducibility. Kidney Int..

[11] Herzenberg A.M. (2011). Validation of the Oxford classification of IgA nephropathy. Kidney Int..

[12] Coppo R. (2014). Validation of the Oxford classification of IgA nephropathy in cohorts with different presentations and treatments. Kidney Int..

[13] Abe T. (1986). Participation of extracapillary lesions (ECL) in progression of IgA nephropathy. Clin. Nephrol..

[14] Lv J. (2013). Evaluation of the Oxford Classification of IgA nephropathy: A systematic review and meta-analysis. Am. J. Kidney Dis..

[15] Lai K.N. (2016). IgA nephropathy. Nat. Rev. Dis. Prim..

[16] Suzuki H. (2009). Aberrantly glycosylated IgA1 in IgA nephropathy patients is recognized by IgG antibodies with restricted heterogeneity. J. Clin. Investig..

[17] Mestecky J. (1993). Defective galactosylation and clearance of IgA1 molecules as a possible etiopathogenic factor in IgA nephropathy. Contrib. Nephrol..

[18] Aryal R.P., Ju T., Cummings R.D. (2010). The endoplasmic reticulum chaperone Cosmc directly promotes in vitro folding of T.-synthase. J. Biol. Chem..

[19] Qin W. (2008). External suppression causes the low expression of the Cosmc gene in IgA nephropathy. Nephrol. Dial. Transpl..

[20] Szeto C.C, Li P.K. (2014). MicroRNAs in IgA nephropathy. Nat. Rev. Nephrol..

[21] Ju T., Cummings R.D. (2002). A unique molecular chaperone Cosmc required for activity of the mammalian core 1 beta 3-galactosyltransferase. Proc. Natl. Acad. Sci. USA.

[22] Leung J.C. (2001). Charge-dependent binding of polymeric IgA1 to human mesangial cells in IgA nephropathy. Kidney Int..

[23] Gharavi A.G. (2008). Aberrant IgA1 glycosylation is inherited in familial and sporadic IgA nephropathy. J. Am. Soc. Nephrol..

[24] Chen X. (2014). Expression and correlation analysis of IL-4, IFN-gamma and FcalphaRI in tonsillar mononuclear cells in patients with IgA nephropathy. Cell Immunol..

[25] Akira S. (2003). Mammalian Toll-like receptors. Curr. Opin. Immunol..

[26] Takeda K., Kaisho T., Akira S. (2003). Toll-like receptors. Annu. Rev. Immunol..

[27] Timmerman I. (2016). Chapter Five—Leukocytes Crossing the Endothelium: A Matter of Communication. International Review of Cell and Molecular Biology.

[28] Reily C. (2014). Cellular Signaling and Production of Galactose-Deficient IgA1 in IgA Nephropathy, an Autoimmune Disease. J. Immunol. Res..

[29] Suzuki H. (2014). Cytokines Alter IgA1 O-Glycosylation by Dysregulating C1GalT1 and ST6GalNAc-II Enzymes. J. Biol. Chem..

[30] Heinrich P.C. (2003). Principles of interleukin (IL)-6-type cytokine signalling and its regulation. Biochem. J..

[31] Wyatt R.J., Julian B.A. (2013). IgA Nephropathy. New Eng. J. Med..

[32] Ng L.G. (2004). B cell-activating factor belonging to the TNF family (BAFF)-R is the principal BAFF receptor facilitating BAFF costimulation of circulating T and B cells. J. Immunol..

[33] Mackay F., Browning J.L. (2002). *BAFF:* A fundamental survival factor for B cells. Nat. Rev. Immunol..

[34] Scapini P., Bazzoni F., Cassatella M.A. (2008). Regulation of B-cell-activating factor (BAFF)/B lymphocyte stimulator (BLyS) expression in human neutrophils. Immunol. Lett..

[35] Zhai Y.L. (2016). Increased APRIL Expression Induces IgA1 Aberrant Glycosylation in IgA Nephropathy. Medicine (Baltimore).

[36] Kim Y.G. (2015). Pathogenic Role of a Proliferation-Inducing Ligand (APRIL) in Murine IgA Nephropathy. PLoS ONE.

[37] Gharavi A.G. (2011). Genome-wide association study identifies susceptibility loci for IgA nephropathy. Nat. Genet..

[38] Xin G. (2013). Serum BAFF is elevated in patients with IgA nephropathy and associated with clinical and histopathological features. J. Nephrol..

[39] McCarthy D.D. (2011). Mice overexpressing BAFF develop a commensal flora-dependent, IgA-associated nephropathy. J. Clin. Investig..

[40] Kiryluk K. (2011). Aberrant glycosylation of IgA1 is inherited in both pediatric IgA nephropathy and Henoch-Schonlein purpura nephritis. Kidney Int..

[41] Tomana M. (1999). Circulating immune complexes in IgA nephropathy consist of IgA1 with galactose-deficient hinge region and antiglycan antibodies. J. Clin. Investig..

[42] Allen A.C. (1998). Abnormal IgA glycosylation in Henoch-Schonlein purpura restricted to patients with clinical nephritis. Nephrol. Dial. Transplant..

[43] Levinsky R.J., Barratt T.M. (1979). IgA immune complexes in Henoch-Schonlein purpura. Lancet.

[44] Lau K.K. (2007). Serum levels of galactose-deficient IgA in children with IgA nephropathy and Henoch-Schonlein purpura. Pediatr. Nephrol..

[45] Suzuki H. (2007). IgA nephropathy: Characterization of IgG antibodies specific for galactose-deficient IgA1. Contrib. Nephrol..

[46] Tomana M. (1997). Galactose-deficient IgA1 in sera of IgA nephropathy patients is present in complexes with IgG. Kidney Int..

[47] Suzuki H. (2008). IgA1-secreting cell lines from patients with IgA nephropathy produce aberrantly glycosylated IgA1. J. Clin. Investig..

[48] Lai K.N. (2012). Pathogenesis of IgA nephropathy. Nat. Rev. Nephrol..

[49] Berthoux F. (2012). Autoantibodies targeting galactose-deficient IgA1 associate with progression of IgA nephropathy. J. Am. Soc. Nephrol..

[50] Novak J. (2008). IgA glycosylation and IgA immune complexes in the pathogenesis of IgA nephropathy. Semin. Nephrol..

[51] Feehally J. (1986). Sequential study of the IgA system in relapsing IgA nephropathy. Kidney Int..

[52] Lai K.N. (2008). Activation of podocytes by mesangial-derived TNF-alpha: Glomerulo-podocytic communication in IgA nephropathy. Am. J. Physiol. Renal. Physiol..

[53] Chan L.Y. (2005). Activation of tubular epithelial cells by mesangial-derived TNF-alpha: Glomerulotubular communication in IgA nephropathy. Kidney Int..

[54] Tam K.Y. (2009). Macromolecular IgA1 taken from patients with familial IgA nephropathy or their asymptomatic relatives have higher reactivity to mesangial cells in vitro. Kidney Int..

[55] Lai K.N. (2009). Podocyte injury induced by mesangial-derived cytokines in IgA nephropathy. Nephrol. Dial. Transplant..

[56] Lai K.N. (2004). Mesangial expression of angiotensin II receptor in IgA nephropathy and its regulation by polymeric IgA1. Kidney Int..

[57] Wang L., Flannery P.J., Spurney R.F. (2003). Characterization of angiotensin II-receptor subtypes in podocytes. J. Lab. Clin. Med..

[58] Chan L.Y. (2005). Tubular expression of angiotensin II receptors and their regulation in IgA nephropathy. J. Am. Soc. Nephrol..

[59] Van Kooten C., Daha M.R., Van Es L.A. (1999). Tubular epithelial cells: A critical cell type in the regulation of renal inflammatory processes. Exp. Nephrol..

[60] Wünsche C. (2015). Transforming growth factor β2 (TGF-β2)-induced connective tissue growth factor (CTGF) expression requires sphingosine 1-phosphate receptor 5 (S1P5) in human mesangial cells. BBA—Mol. Cell Biol. Lipids.

[61] Castro N.E. (2014). Transforming growth factor beta1 (TGF-beta1) enhances expression of profibrotic genes through a novel signaling cascade and microRNAs in renal mesangial cells. J. Biol. Chem..

[62] Rodrigues J.C., Haas M., Reich H.N. (2017). IgA Nephropathy. Clin. J. Am. Soc. Nephrol..

[63] Jennette J.C. (1988). The immunohistology of IgA nephropathy. Am. J. Kidney Dis..

[64] Cooper N.R. (1985). The classical complement pathway: Activation and regulation of the first complement component. Adv. Immunol..

[65] Ferreira V. (2004). The Classical Activation Pathway of the Human Complement System Is Specifically Inhibited by Calreticulin from Trypanosoma cruzi. J. Immunol..

[66] Fishelson Z., Pangburn M.K., Muller-Eberhard H.J. (1984). Characterization of the initial C3 convertase of the alternative pathway of human complement. J. Immunol..

[67] Petersen S.V., Thiel S., Jensenius J.C. (2001). The mannan-binding lectin pathway of complement activation: Biology and disease association. Mol. Immunol..

[68] Sahu A., Lambris J.D. (2001). Structure and biology of complement protein C3, a connecting link between innate and acquired immunity. Immunol. Rev..

[69] Kiryluk K. (2014). Discovery of new risk loci for IgA nephropathy implicates genes involved in immunity against intestinal pathogens. Nat. Genet..

[70] Zhu L. (2015). Variants in Complement Factor H and Complement Factor H-Related Protein Genes, CFHR3 and CFHR1, Affect Complement Activation in IgA Nephropathy. J. Am. Soc. Nephrol..

[71] Rauterberg E.W. (1987). Complement membrane attack (MAC) in idiopathic IgA-glomerulonephritis. Kidney Int..

[72] Miyamoto H. (1988). Immunohistochemical study of the membrane attack complex of complement in IgA nephropathy. Virchows Arch. A. Pathol. Anat. Histopathol..

[73] Onda K. (2011). Excretion of complement proteins and its activation marker C5b-9 in IgA nephropathy in relation to renal function. BMC Nephrol..

[74] Nangaku M., Shankland S.J., Couser W.G. (2005). Cellular response to injury in membranous nephropathy. J. Am. Soc. Nephrol..

[75] Cybulsky A.V. (2000). Complement-induced phospholipase A2 activation in experimental membranous nephropathy. Kidney Int..

[76] Qiu W. (2012). Sublytic C5b-9 complexes induce proliferative changes of glomerular mesangial cells in rat Thy-1 nephritis through TRAF6-mediated PI3K-dependent Akt1 activation. J. Pathol..

[77] Qiu W. (2014). Sublytic C5b-9 triggers glomerular mesangial cell apoptosis via XAF1 gene activation mediated by p300-dependent IRF-1 acetylation. Cell Death Dis..

[78] Komatsu H. (2004). Relationship between serum IgA/C3 ratio and progression of IgA nephropathy. Intern. Med..

[79] Zhang J. (2013). Serum immunoglobulin A/C3 ratio predicts progression of immunoglobulin A nephropathy. Nephrology (Carlton).

[80] Yang P. (2018). Comparative Efficacy and Safety of Therapies in IgA Nephropathy: A Network Meta-analysis of Randomized Controlled Trials. Kidney Int. Rep..

[81] Lozano-Maneiro L., Puente-Garcia A. (2015). Renin-Angiotensin-Aldosterone System Blockade in Diabetic Nephropathy. Present Evidences. J. Clin. Med..

[82] Yang C.-Y. (2018). New Insights into the Immune Molecular Regulation of the Pathogenesis of Acute Respiratory Distress Syndrome. Intern. J. Mol. Sci..

[83] Cheng J. (2009). ACEI/ARB therapy for IgA nephropathy: A meta analysis of randomised controlled trials. Int. J. Clin. Pract..

[84] Marchesi C., Paradis P., Schiffrin E.L. (2008). Role of the renin-angiotensin system in vascular inflammation. Trends Pharmacol. Sci..

[85] Han C. (2010). Angiotensin II induces C-reactive protein expression through ERK1/2 and JNK signaling in human aortic endothelial cells. Atherosclerosis.

[86] Simopoulos A.P. (2002). Omega-3 fatty acids in inflammation and autoimmune diseases. J. Am. Coll. Nutr..

[87] Serhan C.N. (2000). Novel functional sets of lipid-derived mediators with antiinflammatory actions generated from omega-3 fatty acids via cyclooxygenase 2-nonsteroidal antiinflammatory drugs and transcellular processing. J. Exp. Med..

[88] Serhan C.N., Chiang N., Van Dyke T.E. (2008). Resolving inflammation: Dual anti-inflammatory and pro-resolution lipid mediators. Nat. Rev. Immunol..

[89] Serhan C.N. (2002). Resolvins: A family of bioactive products of omega-3 fatty acid transformation circuits initiated by aspirin treatment that counter proinflammation signals. J. Exp. Med..

[90] Donadio J.V., Grande J.P. (2004). The role of fish oil/omega-3 fatty acids in the treatment of IgA nephropathy. Semin. Nephrol..

[91] Reid S. (2011). Non-immunosuppressive treatment for IgA nephropathy. Cochrane Database Syst. Rev..

[92] Greaves M.W. (1976). Anti-inflammatory action of corticosteroids. Postgrad Med. J..

[93] Rhen T., Cidlowski J.A. (2005). Antiinflammatory Action of Glucocorticoids—New Mechanisms for Old Drugs. New Eng. J. Med..

[94] Coppo R. (2018). IgA Nephropathy: A European Perspective in the Corticosteroid Treatment. Kidney Dis. (Basel).

[95] Pozzi C. (2004). Corticosteroid effectiveness in IgA nephropathy: Long-term results of a randomized, controlled trial. J. Am. Soc. Nephrol..

[96] Lv J. (2009). Combination therapy of prednisone and ACE inhibitor versus ACE-inhibitor therapy alone in patients with IgA nephropathy: A randomized controlled trial. Am. J. Kidney Dis..

[97] Manno C. (2009). Randomized controlled clinical trial of corticosteroids plus ACE-inhibitors with long-term follow-up in proteinuric IgA nephropathy. Nephrol. Dial. Transplant..

[98] Ballardie F.W., Roberts I.S. (2002). Controlled prospective trial of prednisolone and cytotoxics in progressive IgA nephropathy. J. Am. Soc. Nephrol..

[99] Pozzi C. (2010). Addition of azathioprine to corticosteroids does not benefit patients with IgA nephropathy. J. Am. Soc. Nephrol..

[100] Pozzi C. (2013). IgA nephropathy with severe chronic renal failure: A randomized controlled trial of corticosteroids and azathioprine. J. Nephrol..

[101] Smerud H.K. (2011). New treatment for IgA nephropathy: Enteric budesonide targeted to the ileocecal region ameliorates proteinuria. Nephrol. Dial. Transplant..

[102] Allison A.C., Eugui E.M. (2000). Mycophenolate mofetil and its mechanisms of action. Immunopharmacology.

[103] Kang Z. (2015). Mycophenolate mofetil therapy for steroid-resistant IgA nephropathy with the nephrotic syndrome in children. Pediatr. Nephrol..

[104] Tan C.H. (2008). Mycophenolate mofetil in the treatment of IgA nephropathy: A systematic review. Singap. Med. J..

[105] Kim M.J. (2012). Spleen tyrosine kinase is important in the production of proinflammatory cytokines and cell proliferation in human mesangial cells following stimulation with IgA1 isolated from IgA nephropathy patients. J. Immunol..

[106] Du B. (2017). Efficacy and safety of mycophenolate mofetil in patients with IgA nephropathy: An update meta-analysis. BMC Nephrol..

[107] Tang S.C. (2010). Long-term study of mycophenolate mofetil treatment in IgA nephropathy. Kidney Int..

[108] Tang S. (2005). Mycophenolate mofetil alleviates persistent proteinuria in IgA nephropathy. Kidney Int..

[109] Appel G.B. (2009). Mycophenolate mofetil versus cyclophosphamide for induction treatment of lupus nephritis. J. Am. Soc. Nephrol..

[110] Hogg R.J. (2015). Randomized controlled trial of mycophenolate mofetil in children, adolescents, and adults with IgA nephropathy. Am. J. Kidney Dis..

[111] Beck L. (2013). KDOQI US commentary on the 2012 KDIGO clinical practice guideline for glomerulonephritis. Am. J. Kidney Dis..

[112] Zhang J. (2001). Cutting Edge: A Role for B Lymphocyte Stimulator in Systemic Lupus Erythematosus. J. Immunol..

[113] US National Library of Science (2018). Safety and Efficacy Study of Fostamatinib to Treat Immunoglobin A (IgA) Nephropathy.

